# Can Albumin/Lymphocyte Ratio and MPV/Lymphocyte Ratio Serve as New Inflammatory Biomarkers in Patients With Hyperemesis Gravidarum?

**DOI:** 10.1002/jcla.70129

**Published:** 2025-11-29

**Authors:** Deniz Taşkıran, Orhan Ay

**Affiliations:** ^1^ Faculty of Medicine Giresun University Giresun Turkey; ^2^ Faculty of Medicine Necmettin Erbakan University Konya Turkey

**Keywords:** albumin/lymphocyte ratio, hyperemesis gravidarum, inflammation, MPV/lymphocyte ratio, systemic inflammatory markers

## Abstract

**Objective:**

This study aimed to evaluate the diagnostic utility of the albumin/lymphocyte ratio (ALR) and mean platelet volume/lymphocyte ratio (MPVLR) novel inflammatory markers not previously studied in hyperemesis gravidarum (HG) as potential biomarkers in patients diagnosed with HG.

**Patients and Methods:**

A total of 68 first‐trimester pregnant women with clinically diagnosed HG and 78 healthy pregnant controls were included. Systemic inflammatory markers including neutrophil/lymphocyte ratio (NLR), platelet/lymphocyte ratio (PLR), albumin/fibrinogen ratio (AFR), fibrinogen/lymphocyte ratio (FLR), magnesium/lymphocyte ratio (MgLR), ALR, MPVLR, and systemic immune‐inflammation index (SII) were calculated and compared between the two groups.

**Results:**

Lymphocyte counts and ketonuria levels were significantly higher in the HG group. Conversely, ALR and MPVLR values were significantly lower in the HG group. MPVLR was identified as a potential diagnostic marker for HG with a cutoff value of 4.569, yielding a sensitivity of 54.4% and specificity of 44.9%. ALR demonstrated a cutoff value of 22.853 with a sensitivity of 61.8% and specificity of 38.5%.

**Conclusions:**

In contrast to the previous literature, PLR and NLR levels were not elevated in HG patients. However, ALR and MPVLR previously unexamined in this population were significantly lower, suggesting a potential role in HG pathophysiology. The findings underscore the complexity and dynamic nature of inflammatory responses in HG, warranting further investigation.

## Graphical abstract

The development of new parameters related to the inflammatory processes of hyperemesis gravidarum will be important for the diagnosis and treatment of the disease. We believe that our study results are important for adding Albumin/Lymphocyte ratio and MPV/Lymphocyte ratio, which have not been previously investigated in hyperemesis gravidarum patients, to the literature.

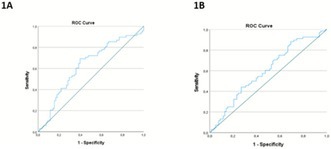

## 1 Introduction

Nausea and vomiting are common features of early pregnancy. Hyperemesis gravidarum (HG) represents a severe clinical form characterized by persistent nausea and vomiting, electrolyte imbalance, nutritional deficiency, and ketonuria. A recent consensus defines HG as severe nausea and vomiting before 16 weeks of gestation, accompanied by reduced oral intake, dehydration, and inability to perform daily activities [1].

While the precise etiology of HG remains unclear, several contributing factors have been proposed, including elevated human chorionic gonadotropin (hCG) levels, thyroid dysfunction, *Helicobacter pylori* infection, immune dysregulation, and inflammatory responses. Numerous studies have explored the role of systemic inflammation in HG, examining markers such as white blood cell (WBC) count, monocyte/lymphocyte ratio (MLR), and C‐reactive protein (CRP), which have been shown to be elevated in HG patients [2, 3]. Similarly, changes in the albumin/fibrinogen ratio (AFR) have been associated with systemic inflammation [4].

Inflammation is known to have an effect on more than one cell. The inflammatory cascade is a very complex and dynamic process. In order to reveal the relationship between inflammatory events and HG, different cellular changes and ratios have been used to explain the pathophysiology of the diseases.

In our study, systemic inflammatory markers were evaluated to assess whether there was a difference between HG patients and women with normal pregnancies. We tried to determine whether fibrinogen, albumin values, and other hemogram parameters, which are simple and rapid oxidative stress markers, can be used in the diagnosis of HG patients. In addition to the inflammatory parameters thought to be effective in the etiology of HG, we tried to evaluate the albumin/fibrinogen ratio, which is a parameter that has not been studied before in this patient group, and to reveal whether it is important for the diagnosis of the disease.

## 2 Materials and Methods

Our study was conducted by retrospectively reviewing the data of pregnant women admitted to a tertiary education and research hospital between October 1, 2023, and April 1, 2024. Sixty‐eight pregnant women with nausea and vomiting in the first trimester and diagnosed as Hyperemesis Gravidarum (HG) and 78 pregnant women without active complaints were included in the study. In our clinic, the diagnosis of hyperemesis gravidarum (HG) is routinely assessed and established based on the PUQE (Pregnancy‐Unique Quantification of Emesis) scoring system. According to the PUQE score, a total score of 4–6 is considered mild, 7–12 moderate, and 13 or above is classified as severe HG. Pregnant women with diabetes mellitus, thyroid disease, urinary tract infection, iron deficiency anemia, multiple pregnancies, and pregnancies outside the first trimester were excluded from the study.

Approval for the study was obtained from the institutional ethics committee with decision number BAEK‐91/18.09.2024/02. In patients diagnosed with hyperemesis gravidarum, complete blood count parameters and biochemical parameters were systematically reviewed, and the results at the time of diagnosis were evaluated.

The HG group consisted of first‐trimester pregnant women with a diagnosis of HG through the hospital system, and the control group consisted of pregnant women with a diagnosis of observation of normal pregnancy. Patients' age, gestational week, gravidity, parity, number of abortions, and body mass index were screened through the hospital system. Body mass index was calculated by dividing body weight (kg) by height squared (m^2^). Complete blood count (CBC), biochemistry parameters, and thyroid values routinely obtained in the first trimester were evaluated. Complete blood count (CBC) parameters were measured using Mindray bc 5800 Automated Hematology Analyzer (Mindray, China), an automated blood count machine.

Hemoglobin, platelet, neutrophil, lymphocyte count, mean platelet volume (MPV), mean corpuscular volume (MCV) values were recorded from the CBC records. Albumin, magnesium, and thyroid‐stimulating hormone (TSH) values were recorded. Neutrophil lymphocyte ratio (NLR), platelet lymphocyte ratio (PLR), albumin fibrinogen ratio (AFR), albumin lymphocyte ratio (ALR), fibrinogen lymphocyte ratio (FLR), magnesium lymphocyte ratio (MgLR), MPV lymphocyte ratio (MPVLR), and systemic immune‐inflammation index (SII) values were calculated using these values. Systemic immune‐inflammation index (SII) was calculated by the formula platelet count × neutrophil count/lymphocyte count. Systemic inflammation indices and other parameters were compared between the HG group and first trimester pregnant women without active complaints.

### 2.1 Statistical Analysis

Data analysis was performed using Statistical Package for Social Sciences (SPSS), version 27.0 (IMBCorp., Armonk, NY, USA). *p* < 0.05 was considered statistically significant. The Kolmogorov–Smirnov test was used to assess normality distribution. Continuous variables were presented as mean ± standard deviation (SD) and those not normally distributed were presented as median (minimum–maximum). Groups with abnormal distribution were compared using the Mann–Whitney *U* test and those with normal distribution were compared using the Student's *t*‐test.

## 3 Results

In the comparison between groups, gestational week (8 (6–14) vs. 8 (6–14); *p*: 0.528), body mass index (26.12 ± 5.67 vs. 25.42 ± 4.23; *p*: 0.326), gravidity (2 (1–5) vs. 2 (1–9); *p*: 0.52), parity (0 (0–3) vs. 0 (0–6); *p*: 0.154), and abortion (0 (0–3) vs. 0 (0–4); *p*: 0.353). Among complete blood count and biochemistry parameters, hemoglobin (12.23 ± 0.96 vs. 12.44 ± 1.08; *p*: 0.212), neutrophils (5.98 ± 1.84 vs. 6.0.9 ± 1.71; *p*: 0.618), platelets (254.56 ± 60.39 vs. 258.34 ± 56.47; *p*: 0.476), MCV (85.50 ± 4.67 vs. 83.42 ± 6.93; *p*: 0.093), MPV (9.31 ± 0.98 vs. 9.28 ± 0.97; *p*: 0.863), albumin (44.95 ± 2.81 vs. 44.52 ± 2.79; *p*: 0.213), fibrinogen (344.64 ± 83.57 vs. 353.26 ± 77.87; *p*: 0.494), magnesium (1.93 ± 0.17 vs. 1.96 ± 0.13; *p*: 0.352), and TSH (2.50 ± 1.83 vs. 2.98 ± 7.87; *p*: 0.299) were similar between both groups (Table 1).

**TABLE 1:** Comparison of the results of patients with HG and normal pregnancy in the first trimester.

	Normal pregnant women (*n* = 78)		Hyperemesis gravidarum (*n* = 68)		*p*
	Mean ± SD	Median (Min–Max)	Mean ± SD	Median (Min–Max)	
Age (years)	27.91 ± 4.69	27.5 (20–40)	29.59 ± 5.02	29 (21–43)	**0.035**
Gestation week	8.76 ± 2.25	8 (6–14)	9 ± 2.4	8 (6–14)	0.528
Body Mass Index (BMI) (kg/m^2^)	26.12 ± 5.67	27.03 (24.12‐ 34.15)	25.42 ± 4.23	26.95 (23.89‐ 32.12)	0.326
Gravide	2 ± 1	2 (1–5)	3 ± 1	2 (1–9)	0.520
Parity	1 ± 1	0 (0–3)	1 ± 1	0 (0–6)	0.154
Abortion	0.44 ± 0.74	0 (0–3)	0.34 ± 0.74	0 (0–4)	0.353
Hemoglobin (g/dL)	12.23 ± 0.96	12.25 (9.6‐ 14.3)	12.44 ± 1.08	12.5 (10–14.7)	0.212
Platelets (10^9^/L)	254.56 ± 60.39	241 (149–457)	258.34 ± 56.47	259 (134–387)	0.476
Neutrophil (μL)	5.98 ± 1.84	5.63 (2.38‐ 10.69)	6.09 ± 1.71	5.68 (2.82‐ 10.9)	0.618
Lymphocytes (μL)	1.98 ± 0.92	1.84 (1.13‐ 8.6)	2.11 ± 0.57	2 (0.93‐ 3.76)	**0.017**
Mean erythrocyte volume (MCV) (fL)	85.50 ± 4.67	85.11 (73.4–96)	83.42 ± 6.93	84.05 (62.8‐95.4)	0.093
Mean platelet volume (MPV) (pg)	9.31 ± 0.98	9.25 (7.7‐ 12.5)	9.28 ± 0.97	9.11 (7.7‐11.5)	0.863
Albumin (g/DL)	44.95 ± 2.81	45.5 (38–51.9)	44.52 ± 2.79	44.65 (37.8‐ 53.4)	0.213
Fibrinogen (mg/dL)	344.64 ± 83.57	338 (134–575)	353.26 ± 77.87	352 (200–528)	0.494
Magnesium (mEq/L)	1.93 ± 0.17	1.95 (1–2.23)	1.96 ± 0.13	1.97 (1.73‐2.24)	0.352
Thyroid stimulating hormone (TSH) (mUl/mL)	2.50 ± 1.83	1.86 (0.39–9)	2.98 ± 7.87	1.86 (0.08–66)	0.299
Ketone in urine	0.08 ± 0.26	0 (0–1)	1.34 ± 0.97	1 (0–3)	**0.001**

*Note:* Mann–Whitney *U* test, Student's *t*‐test (*p* < 0.05).

Abbreviations: max, maximum; min, minimum; SD, standard deviation.

The mean age (29.59 ± 5.02 vs. 27.91 ± 4.69; *p*: 0.035) and urine ketone positivity (1.34 ± 0.97 vs. 0.08 ± 0.26; *p*: 0.001) were higher in the HG group (Table 1).

When normal pregnancy and HG groups were compared, NLR (3.19 ± 0.84 vs. 3.04 ± 1.01; *p*: 0.243), PLR (138.81 ± 39.21 vs. 129.21 ± 39.66; *p*: 0.076), AFR (0.13 ± 0.04 vs. 0.13 ± 0.03; *p*: 0.419), FLR (192.67 ± 66.79 vs. 179.42 ± 61.61; *p*: 0.149), MgLR (1.08 ± 0.30 vs. 1 ± 0.29; *p*: 0.053), and SII (807.25 ± 294.67 vs. 781.91 ± 303.05; *p*: 0.583) (Table 2).

**TABLE 2:** Comparison of inflammatory parameters of patients with HG and normal pregnancy in the first trimester.

	Normal pregnant women (*n* = 78)		Hyperemesis gravidarum (*n* = 68)		*p*
	Mean ± SD	Median (Min–Max)	Mean ± SD	Median (Min–Max)	
Neutrophil/Lymphocyte ratio (NLR)	3.19 ± 0.84	3.11 (1.76–5.51)	3.04 ± 1.01	3.01 (0.96–5.90)	0.243
Platelet/Lymphocyte ratio (PLR)	138.81 ± 39.21	136.31 (29.88– 243.65)	129.21 ± 39.66	125.01 (51.34– 260.91)	0.076
Albumin/Fibrinogen ratio (AFR)	0.13 ± 0.04	0.13 (0.08 – 0.35)	0.13 ± 0.03	0.12 (0.08–0.24)	0.419
Fibrinogen/Lymphocyte ratio (FLR)	192.67 ± 66.79	192.12 (32.33– 366.37)	179.42 ± 61.61	176.78 (79.38– 340.86)	0.149
Magnesium/Lymphocyte ratio (MgLR)	1.08 ± 0.30	1.06 (0.25– 1.69)	1 ± 0.29	0.95 (0.48– 2.06)	0.053
Albumin/Lymphocyte ratio (ALR)	25.06 ± 6.73	25.03 (5.44–39.91)	22.78 ± 7.16	21.59 (10.05– 46.99)	**0.009**
MPV/Lymphocyte ratio (MPVLR)	5.25 ± 1.69	4.67 (1–9.39)	4.74 ± 1.47	4.44 (2.07–9.25)	**0.046**
Systemic Immune‐Inflammation Index (SII)	807.25 ± 294.67	719.92 (254.01– 1867.17)	781.91 ± 303.05	719.32 (183.8–1773.46)	0.583

*Note:* Mann–Whitney *U* test, Student's *t*‐test (*p* < 0.05).

Abbreviations: max, maximum; min, minimum; SD, standard deviation.

**FIGURE 1:**
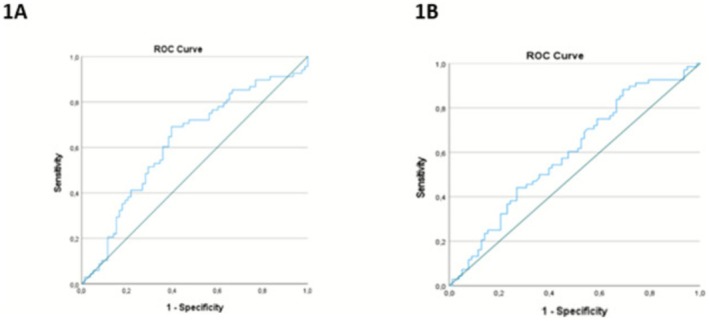
(A) ROC curve for the diagnostic value of ALR index in determining the presence and severity of hyperemesis gravidarum (HG). (B) ROC curve for the diagnostic value of the MPVLR index in determining the presence and severity of hyperemesis gravidarum (HG).

When normal pregnancy and HG groups were compared, ALR (25.06 ± 6.73 vs. 22.78 ± 7.16; *p*: 0.009) and MPVLR (5.25 ± 1.69 vs. 4.74 ± 1.47; *p*: 0.046) values were higher in the normal pregnancy group (Table 2). ALR and MPVLR, ROC curves are shown in Figure 1A,B. The ALR index was used as a diagnostic evaluation tool in the presence of hyperemesis gravidarum (HG), with a cutoff value of 22.85, demonstrating a sensitivity of 61.8% and a specificity ofPage 17 of 36 38.5%. The area under the curve (AUC) was calculated as 0.625. Similarly, the MPVLR index was determined as a diagnostic marker in HG with a cutoff value of 4.56, showing a sensitivity of 54.4% and a specificity of 44.9%, with an AUC of 0.596. The cutoff values of ALR and MPVLR indices are shown in Table 3.

**TABLE 3:** Sensitivity and specificity values of the ALR and MPVLR biomarkers.

	AUC (%95)	Cutoff	*p*	Sensitivity (%)	Specificity (%)
ALR	0.625 (0.533–0.717)	22.853	**0.009**	61.8	38.5
MPVLR	0.596 (0.504–0.688)	4.569	**0.046**	54.4	44.9

*Note:* MPVLR index was determined as a diagnostic evaluation tool in the presence of HG with a cutoff value of 4.56 with a sensitivity of 54.4% and a specificity of 44.9% (Figure 1A,B). ALR index was determined as a diagnostic evaluation tool with a sensitivity of 61.8% and specificity of 38.5% with a cut‐off value of 22.85 in the presence of HG.

## 4 Discussion

HG is a clinical condition with unclear pathophysiology and inflammation is thought to have a significant contribution [5]. Pregnancy hormones, gastrointestinal dysmotility, nutritional disorders, and immunologic causes have been blamed in the pathophysiology. In recent years, the effect of inflammation on HG has become more accepted. The aim of our study was to reveal the relationship between the presence of HG and inflammatory markers in patients diagnosed with HG and to contribute to the literature.

The results of our study revealed that the lymphocyte ratio was higher in patients with HG. NLR, PLR, AFR, MgLR, and SII, which are thought to be important in systemic inflammatory response, were similar in HG and normal pregnant women. Advanced gestational age was shown to be a non‐modifiable risk factor for HG. ALR and MPVLR, indicators of systemic inflammatory response, were found to be lower in HG patients. These results suggest that HG induced systemic inflammatory response may not be as effective as expected at the cellular level. In addition, although there are many studies on NLR and PLR in HG, the fact that Fibrinogen/Lymphocyte ratio (FLR), Magnesium/Lymphocyte ratio (MgLR), Albumin/Lymphocyte ratio (ALR), and MPV/Lymphocyte ratio (MPVLR) indices have not been adequately studied in HG patients will contribute to the literature.

Pregnancy nausea and vomiting affects approximately 50%–80% of women [6]. In HG, which is a more severe form of nausea and vomiting, weight loss, fluid electrolyte imbalance, and malnutrition are observed in patients. Clinical signs of HG begin to be observed in the early first trimester and end approximately at the 20th gestational week. In some pregnant women, persistent attacks of nausea and vomiting continue throughout pregnancy [7]. In addition to severe weight loss, dehydration, and vitamin K deficiency, severe morbidity such as life threatening Wernicke's encephalopathy and central pontine myelinosis can be observed [8].

The high levels of CRP and IL‐6 in HG patients suggest that inflammation may be effective in the pathogenesis of Hg [9]. A number of changes occur in the maternal immune system for the maintenance of a healthy pregnancy. It is known that granulocyte, natural killer cells, and extrathymic cell activation occur in pregnancy [10]. Neutrophils constitute 50%–70% of all white blood cells and have an average lifespan of 5.4 days due to oxidative stress response [11]. Neutrophil production and degradation processes may be affected by HG induced inflammatory processes, which may alter the NLR rate. The use of inflammation marker parameters such as NLR and PLR in the diagnosis of acute inflammatory diseases has been shown to be a low‐cost diagnostic tool [12]. It is known that NLR and PLR have clinical use in the evaluation of the inflammatory response in systemic lupus erythematosus [13]. In a study, it was reported that the NLR and PLR ratio was high in patients with HG [14]. It has been reported that the use of NLR as an inflammatory marker in acute coronary syndrome is a cost effective marker and may provide information about the clinical predictability and prognosis of patients [15]. The use of NLR and PLR as inflammatory markers in gastric cancer has been shown to be more significant than tumor markers as systemic inflammatory markers [16]. However, in our study, the NLR and PLR ratios in the HG group were found to be similar to the normal pregnant group. Since the construction and destruction of cells is a dynamic process, it may have been similar in both groups. Contrary to the studies, it may be misleading to comment on PLR and NLR ratios in HG patients.

It is thought that cell concentration will increase in HG due to dehydration caused by severe vomiting. Accordingly, studies have shown that the number of lymphocytes is higher in HG [17]. However, there are conflicting data on lymphocyte count in the literature. In our study, lymphocyte count was found to be higher in the HG group and hemoglobin, platelet and neutrophil counts were similar in both groups.

MPV is used as a marker of platelet activation in the diagnosis of inflammatory diseases [18]. MPV is one of the most widely studied markers of platelet activation. Increased platelet activation due to inflammation and an increase in MPV ratio are expected. There are different results in the literature regarding MPV values of patients diagnosed with HG. When its ratio with lymphocyte count is evaluated, it is thought that it may allow us to comment on inflammation processes. MPVLR was studied for the first time in HG patients. In our study, the MPVLR ratio was found to be low in the HG group and this may be due to the increase in lymphocyte count in HG patients. In the ROC analysis, it was determined that MPVLR could be used for the diagnosis of HG in first‐trimester pregnant women with a cutoff value of 4.569 with a sensitivity of 54.4% and a specificity of 44.9%.

Pregnancy and hypoalbuminemia due to nausea and vomiting and inflammation may cause a decrease in ALR. A simple and inexpensive assessment of the ALR ratio may be important for the diagnosis of HG. ALR in HG has not been studied before. In our study, we found that ALR can be used for the diagnosis of HG in first trimester‐pregnant women with a cutoff value of 22.853 with 61.8% sensitivity and 38.5% specificity.

Regardless of the severity of HG, ketonuria is commonly used for the diagnosis of HG. The ketonuria value is used to monitor metabolic outcomes and clinical recovery of patients. It has been shown that the ketonuria level is associated with the length of hospitalization [19]. In our study, ketone positivity was shown to support the diagnosis of HG.

The limitations of our study include the retrospective nature of the study and the fact that the diagnosis of HG was based on clinical findings without any questionnaire.

## 5 Conclusion

Contrary to the literature, our study showed that PLR and NLR were not high in HG patients, while ALR and MPVLR, which have not been previously studied, were lower in HG patients. It shows that the effect of inflammatory processes in the pathophysiology of HG is not clear. The fact that the inflammatory processes in HG are complex and the cells are in a dynamic process suggests that further studies are needed.

## Author Contributions

**Deniz Taşkıran:** writing – review and editing, conceptualization, writing – original draft preparation, data curation, project administration, supervision. **Orhan Ay:** writing – review and editing, validation.

## Disclosure

The authors confirm that the ethical policies of the journal, as noted on the journal's author guidelines page, have been adhered to and the appropriate ethical review committee approval has been received. The study was conducted in accordance with the Helsinki declaration.

## Conflicts of Interest

The authors declare no conflicts of interest.

## Data Availability

The data that support the findings of this study are available upon request from the corresponding author. The data are not publicly available due to privacy or ethical restrictions.
